# Cartilage Injuries of the Ankle: New Beginnings

**DOI:** 10.1177/19476035251361353

**Published:** 2025-07-24

**Authors:** Gino M.M.J. Kerkhoffs, John G. Kennedy, Mats Brittberg, Jari Dahmen

Dear Colleagues,

We extend a warm welcome to the inaugural special issue of the *CARTILAGE* Journal focused on cartilage injuries of the ankle.

Over the last decades, we have come to understand more and more that ankle cartilage issues are frequent and can arise even from a not-so-simple lateral ankle sprain or low-velocity ankle fracture. This potentially causes a domino effect or cascade of progressive cartilage damage.^1-4^ Time has shown that the sequelae of ankle cartilage damage increasingly impact the quality of life of our patients: chronic deep ankle pain, restricted mobility, persistent swelling, and an overall decline in well-being are frequently reported symptoms.^5-7^ These ankle cartilage problems, particularly when they are not addressed adequately in the early stages, also may have the tendency to progress to the end-stage, ankle osteoarthritis.^2^ At this critical juncture, treatment options are limited to either ankle joint fusion or total ankle replacement (TAR).^8-11^ Unfortunately, the outcomes of TAR tend to be less favorable when compared to those of total hip or knee prostheses.^12,13^ Hence, in this special issue, we will focus on improving our understanding around the development of ankle cartilage damage. Moreover, we will highlight the need for a further standardization of reporting of earlier-stage cartilage lesions and discuss several aspects of different treatment options for ankle osteochondral lesions.

Recent research has improved our understanding of the early stages of cartilage damage to allow for improved prevention and treatment strategies. For instance, in this issue, Blom *et al.*^14^ present a study on micro-indentation that a single traumatic impact on the ankle can cause an immediate and substantial decrease in the storage and loss of moduli of the cartilage. This change suggests a potential rupture of collagen fibers, which disrupts the cartilage’s ability to bind water-binding proteoglycans and reduces the hydrostatic pressure that is critical for cartilage resilience.^15,16^ This finding emphasizes the need for innovative, non-invasive diagnostic tools to detect early cartilage damage and to monitor its progression to post-traumatic osteoarthritis (PTOA).

Shifting toward the treatment of cartilage and osteochondral lesions of the ankle, treatment options for osteochondral lesions of the talus (OLT) are currently evolving rapidly.^7,17^ As such, there is a growing need for a clear standardization in diagnostic and prognostic reporting. In the study by van Diepen *et al.*,^18^ the authors highlight the lack of consensus on the classification and reporting of lesion morphology, location, and size. This inconsistency may complicate decision-making and underscores the need for a universally accepted classification system, such as a validated OLT classification system. Such a new system will ameliorate the communication around the classification of osteochondral lesions of the ankle, eventually also having an effect on the harmonization of research and clinical approaches.

We also focus on the conservative treatment of OLT. Buck *et al.*^19^ identify risk factors for the conversion to surgery after initial nonoperative treatment of OLTs. In the retrospective cohort consisting of 42 patients, 77% of the patients are successfully treated non-operatively at a median follow-up of 66 months (i.e., no need for a surgical intervention). A higher age at the moment of diagnosis was significantly associated with a lower likelihood of conversion to surgery. These outcomes can be readily applied in clinical practice and open doors toward further research regarding the effectiveness of nonoperative treatment options for OLTs, also considering longer follow-up times.

In the surgical realm, the use of orthobiologics has extensively been researched and discussed, and there are still many research gaps to move forwards in this field.^20-22^ Butler *et al.*^23^ aim at filling one of these by presenting a prospective study characterizing the macrophage subpopulation within concentrated bone marrow aspirate (cBMA), which may have significant clinical implications in future studies using cBMA in the treatment of ankle cartilage defects.^24^ The authors conclude that this study is the first to characterize macrophage subpopulations within cBMA. Their findings may serve as a foundation for developing targeted cartilage repair therapies by promoting macrophage polarization toward the M2 phenotype.^25,26^

Surgical treatment options such as autograft transfers, in the form of Talar OsteoPeriostic grafting from the Iliac Crest (TOPIC) and Autologous Osteochondral Transplantation (AOT) were also presented.^5,27,28^ The long-term survival after AOT was studied by Butler *et al.*^29^ through a relatively large retrospective cohort. The authors reported a survival rate of 94% for large OLTs treated with AOT at a minimum of 10-year follow-up. For smaller lesions, particularly those confined to the subchondral bone beneath the chondral layer, retrograde drilling may offer a valuable, less invasive treatment option.^30-32^ However, the literature on the clinical outcomes on this treatment option is scarce, as pointed out by Yasui *et al.*,^33^ who conclude in their systematic review that there is a grand research gap on this particular matter. The systematic review by the Japanese author group thereby concludes that more research is warranted with potential future international research collaborations pointing in this direction.

Although clinical outcomes are frequently used as an assessment marker after surgical treatment for chondral and osteochondral lesions of the ankle, the quality of cartilage repair as determined by second-look arthroscopy is also considered an important tool. In a systematic review and meta-analysis, Vreeken *et al.*^34^ compared the success and failure rates of cartilage repair of OLTs as assessed during second-look arthroscopy after various surgical interventions. The author group shows that at second look, the quality of ankle cartilage repair is significantly higher after fixation or autografting procedures when compared to bone marrow stimulation techniques. Further building on the study of Vreeken *et al.*,^34^ another study in this specific field was conducted. Walinga *et al.*^35^ demonstrate the utility of second-look needle arthroscopy for evaluating and treating cartilage damage from an Amsterdam and New York perspective, thereby researching the quality of reparative cartilage at short- to mid-term follow-up after a TOPIC and AOT procedure. The authors conclude that this new needle arthroscopy system to assess quality in a minimally invasive manner was successful and safe, and that the International Cartilage Repair Society (ICRS) scores at follow-up after both the TOPIC and AOT procedures were considered good. This study can serve as a foundation for future research involving larger prospective cohorts, where second-look needle arthroscopy may be used to assess cartilage repair following surgical treatments for (focal) chondral and osteochondral injuries in various joints.

In an internationally oriented author team led by Nakasa *et al.*,^36^ the evidence-based recommendations around fixation of OLT were discussed to provide the readers with an updated approach on how to fix these lesions. For lesions that may not be amenable for fixation, Efrima *et al.*^37^ describe the mid- to long-term clinical outcomes of patients who underwent arthroscopic autologous matrix-induced chondrogenesis (AMIC) for OLT, as currently the research on AMIC for OLTs is relatively minimal.^38-40^ The authors conclude that AMIC is an effective treatment strategy for OLTs with clinical improvement peaking in the first 2 years.

This issue has taken us several steps closer to understanding ankle cartilage damage, while also reminding us that each new insight often uncovers even more questions—particularly in the realms of early detection, prevention of progression, and treatment across the various stages of this condition. We believe the future lies in advancing early diagnostic tools and developing minimally invasive (preventive) interventions that can halt the disease before significant degeneration occurs.


Gino M.M.J. Kerkhoffs Department of Orthopedic Surgery and Sports Medicine, Amsterdam UMC, Location AMC, Amsterdam Movement Sciences, University of Amsterdam, Amsterdam, The Netherlands Amsterdam Collaboration for Health and Safety in Sports (ACHSS), AMC/VUmc IOC Research Center, Amsterdam, The Netherlands Academic Center for Evidence-Based Sports Medicine, Amsterdam UMC, Amsterdam, The Netherlands
John G. Kennedy Department of Orthopaedic Surgery, New York University Langone Health, New York, NY, USA
Mats Brittberg Cartilage Research Unit, Region Halland Orthopaedics, Kungsbacka Hospital, University of Gothenburg, Kungsbacka, Sweden
Jari Dahmen Department of Orthopedic Surgery and Sports Medicine, Amsterdam UMC, Location AMC, Amsterdam Movement Sciences, University of Amsterdam, Amsterdam, The Netherlands Amsterdam Collaboration for Health and Safety in Sports (ACHSS), AMC/VUmc IOC Research Center, Amsterdam, The Netherlands Academic Center for Evidence-Based Sports Medicine, Amsterdam UMC, Amsterdam, The Netherlands

